# Performance of hybrids between abiotic stress-tolerant transgenic rice and its weedy relatives under water-stressed conditions

**DOI:** 10.1038/s41598-020-66206-3

**Published:** 2020-06-09

**Authors:** Kyong-Hee Nam, Do Young Kim, Ye Seul Moon, In Soon Pack, Soon-Chun Jeong, Ho Bang Kim, Chang-Gi Kim

**Affiliations:** 1 LMO research team, National Institute of Ecology, Seocheon, 33657 Republic of Korea; 2 Bio-Evaluation Center, Korea Research Institute of Bioscience & Biotechnology, Cheongju, 28116 Republic of Korea; 3 Life Sciences Research Institute, Biomedic Co., Ltd., Bucheon, 14548 Republic of Korea

**Keywords:** Ecology, Evolution

## Abstract

Gene transfer from transgenic crops to their weedy relatives may introduce undesired ecological consequences that can increase the fitness and invasiveness of weedy populations. Here, we examined the rate of gene flow from abiotic stress-tolerant transgenic rice that over-express *AtCYP78A7*, a gene encoding cytochrome P450 protein, to six weedy rice accessions and compared the phenotypic performance and drought tolerance of their hybrids over generations. The rate of transgene flow from *AtCYP78A7*-overexpressing transgenic to weedy rice varied between 0% and 0.0396%. F_1_ hybrids containing *AtCYP78A7* were significantly taller and heavier, but the percentage of ripened grains, grain numbers and weight per plant were significantly lower than their transgenic and weedy parents. The homozygous and hemizygous F_2_ progeny showed higher tolerance to drought stress than the nullizygous F_2_ progeny, as indicated by leaf rolling scores. Shoot growth of nullizygous F_3_ progeny was significantly greater than weedy rice under water-deficient conditions in a rainout shelter, however, that of homozygous F_3_ progeny was similar to weedy rice, indicating the cost of continuous expression of transgene. Our findings imply that gene flow from *AtCYP78A7*-overexpressing transgenic to weedy rice might increase drought tolerance as shown in the pot experiment, however, increased fitness under stressed conditions in the field were not observed for hybrid progeny containing transgenes.

## Introduction

One of the major environmental concerns with transgenic crops is the transgene movement and proliferation into wild or weedy relatives^1^. Although the rate of gene flow from transgenic crops to their related species is not high, the introgression of a transgene may introduce potential ecological consequences that can elevate the fitness and invasiveness of those weedy/wild populations^2–6^.

The hybrids generated by gene flow from *Bacillus thuringiensis* (*Bt*)-transgenic oilseed rape to its weedy relative, *Brassica rapa*, resulted in an intermediate phenotype between the parental species and synthesized the *Bt* Cry1Ac protein at a similar level to the transgenic oilseed rape lines^7^. Decreases in the tiller numbers per plant, grain numbers per panicles, and percent seed fertility was observed for the hybrids between transgenic herbicide-resistant rice and weedy rice compared to their parental lines^8^ Chun *et al*.^9^ have reported that the homozygous F_2_ progeny of herbicide-resistant transgenic rice and weedy rice were considerably taller and produce more grains per plant compared to their parental transgenic and weedy relatives. Research on hybrids of transgenic crops and weedy relatives, undertaken under laboratory-controlled or field conditions might provide some insight and help predict the possibility of long-term persistence and evolution of hybrids to become more invasive weeds.

Drought is one of the most significant abiotic constraints reducing crop growth and yield worldwide^10^. In response to drought stress, plants often induce the expression of many transcriptional regulators, which in turn, up-regulated a series of downstream genes for stress adaptation or self-protection^11,12^. Furthermore, genetic manipulation for the improvement of drought tolerance includes the regulation of complex multigene networks and may therefore have greater pleiotropic effects than the simple monogenic traits that dominate the global market for plant biotechnology at present^13–15^. For instance, the transgenic expression of *DREB1A*, a single stress-inducible transcription factor that recognizes dehydration-response elements, enhances tolerance not only to drought but also to salt and freezing stresses^16^. Ectopic expression of *GmERF3*, a member of the APETALA2/ethylene response factor (AP2/ERF) transcription factor gene family, promotes tolerance to drought and high salinity, and further increases resistance against the bacterial pathogen *Ralstonia solanacearum*, the fungal pathogen *Alternaria alternata*, and tobacco mosaic virus in transgenic tobacco plants^17^. Accordingly, it is important to investigate the performance of hybrids resulting from gene flow between drought-tolerant transgenic crops and their weedy relatives because a transgene might confer diverse beneficial traits (including drought tolerance) to the hybrids.

It has been reported that the overexpression of *AtCYP78A7*, a gene encoding cytochrome P450 protein, exhibits improved drought tolerance and increased seed size in transgenic rice^18^. Cytochrome P450 monooxygenases are known to be involved in the biosynthesis of numerous secondary metabolites and stress responses of plants to herbicides and pathogens^19–23^. In our previous studies, overexpression of *AtCYP78A7* led to changes in nutritional composition and metabolite profile depending on water conditions of transgenic rice^24,25^. Here, we examined the rate of gene flow from *AtCYP78A7*–overexpressing abiotic stress-tolerant transgenic to weedy rice and then identified the phenotypic performance and drought tolerance in their hybrids and subsequent descendants derived from this transgene flow.

## Results

### Gene flow from AtCYP78A7-overexpressing transgenic rice to weedy rice

The detectable rate of transgene flow from *AtCYP78A7*-overexpressing abiotic stress-tolerant transgenic rice to six weedy rice accessions ranged from 0 to 0.0396% (Table 1). Transgenic hybrids with the *AtCYP78A7* gene were detected with Geojeaengmi 20 (hereinafter “Ge 20”), Gunsanaengmi 1 (hereinafter “Gu 1”), Boeunaengmi 5 (hereinafter “Bo 5”), Muanaengmi 15 (hereinafter “Mu 15”), and Hwaseongaengmi 1 (hereinafter “Hw 1”), but not with Yesanaengmi 3 (hereinafter “Ye 3”). Flowering periods of transgenic donors partially overlapped with those of all recipient weedy accessions; Ye 3 has the shortest overlapping period.

**Table 1:** Heading date and transgene flow rate (%) of six weedy rice accessions (pollen recipients) from transgenic pollen donor.

Weedy rice		First heading date	Heading date	Full heading date	% gene flow rate
Accession no.	Accession name				
YW1434	Geojeaengmi 20 (Ge 20)	4 Aug	12 Aug	17 Aug	0.0128 (5/38,937)
YW1456	Gunsanaengmi 1 (Gu 1)	3 Aug	11 Aug	14 Aug	0.0396 (15/37,837)
YW3009	Boeunaengmi 5 (Bo 5)	15 Aug	19 Aug	24 Aug	0.0054 (1/18,469)
YW3001	Muanaengmi 15 (Mu 15)	12 Aug	15 Aug	17 Aug	0.0042 (2/48,130)
YW2612	Hwaseongaengmi 1 (Hw 1)	7 Aug	11 Aug	13 Aug	0.0044 (1/22,676)
YW2511	Yesanaengmi 3 (Ye 3)	17 Aug	19 Aug	24 Aug	0.0000 (0/34,486)

Values in parentheses are the number of hygromycin-resistant seedlings/number of evaluated seedlings.

First heading date, heading date, and full heading date of AtCYP78A7-overexpressing transgenic rice was 10 Aug, 14 Aug, and 19 Aug, respectively, in 2012.

### Phenotypic performance of F_1_ hybrids

**Figure 1:**
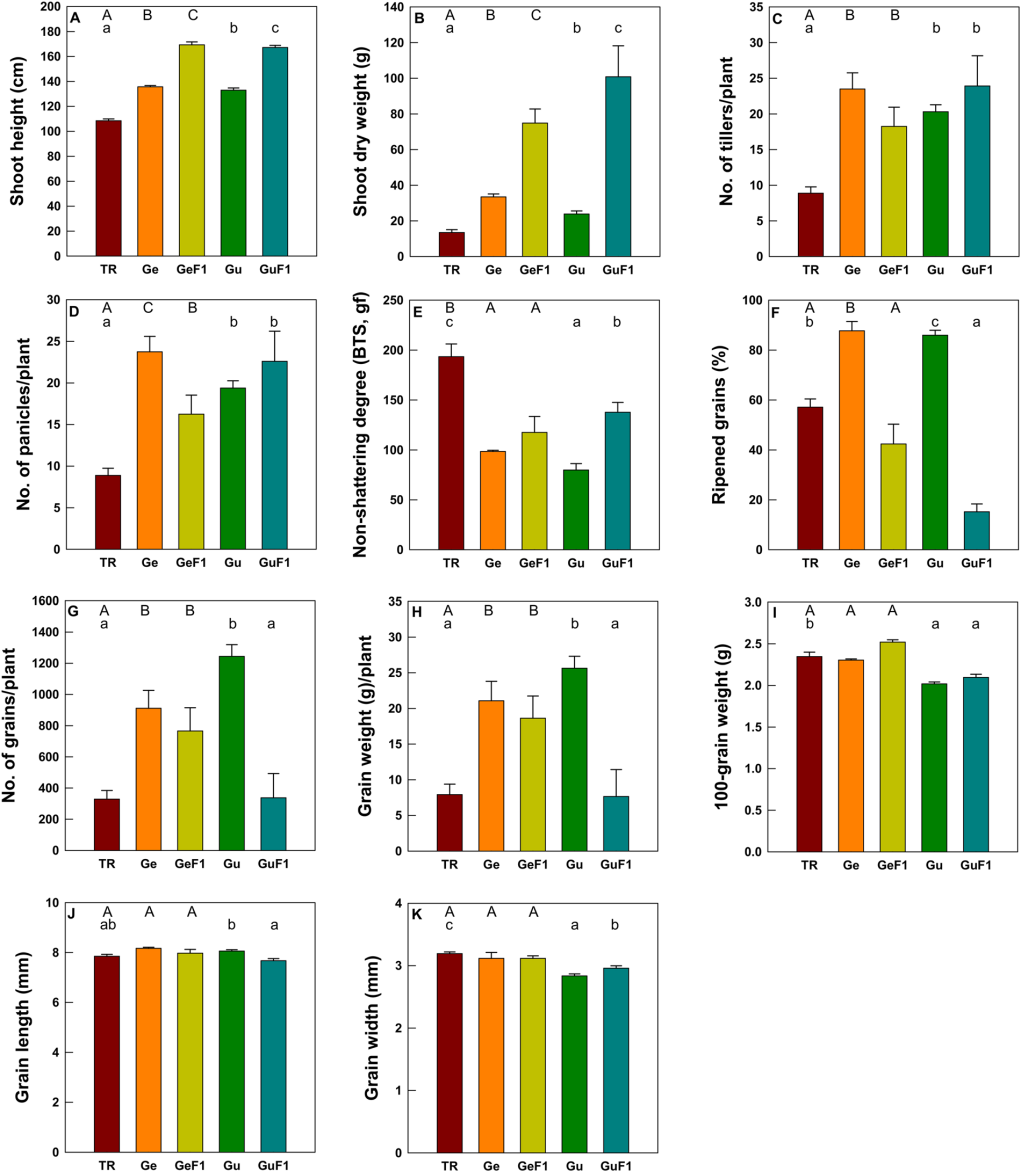
Comparison of phenotypic traits for F_1_ hybrids (Ge 20 × transgenic and Gu 1 × transgenic crosses) between *AtCYP78A7*-overexpressing transgenic rice and two weedy rice accessions, and their parental transgenic and weedy rice. Data are means ± standard errors. TR, transgenic rice; Ge, Ge 20; Gu, Gu 1. Uppercase and lowercase letters are used to indicate significant differences between means of transgenic rice and Ge 20 × transgenic rice cross, and transgenic rice and Gu 1 × transgenic rice cross, respectively (*p* < 0.05).

Among the F_1_ hybrids that resulted from transgene flow, we compared the phenotypic performance of Ge 20 × transgenic and Gu 1 × transgenic crosses with those of their parental transgenic and weedy rice (Fig. 1). Compared with transgenic rice, F_1_ hybrids between transgenic rice and Ge 20 exhibited: i) significantly higher shoot height (56.1%) and shoot biomass (456.2%), tiller (105.3%), panicle (82.8%), and number and weight of grains per plant (133.0% and 135.3%, respectively), and ii) lower non-shattering degree (39.3%). The percentage of ripened grains, 100-grain weight, grain length and weight were not significantly different between transgenic rice and Ge 20 × transgenic F_1_ hybrids. When compared with the Ge 20 weedy parent, the Ge 20 × transgenic F_1_ hybrids were 24.8% taller and 124.0% heavier, but panicle number and percentage of ripened grains were 31.6% and 51.7% lower, respectively. Number of tillers per plant, non-shattering degree, numbers and weight of grains per plant, 100-grain weight, grain length and width was not significantly different between the Ge 20 × transgenic F_1_ hybrids and weedy rice.

The F_1_ hybrids of transgenic and Gu 1 weedy accession crosses had higher shoot height (53.5%) and shoot biomass (437.8%) and larger numbers of tiller (93.3%) and panicle (80.0%) compared with transgenic rice. In contrast, the Gu 1 × transgenic F_1_ hybrids showed lower non-shattering degree (27.3%), percentage of ripened grains (78.8%), 100-grain weight (11.5%), and grain width (7.8%) compared with transgenic rice. Grain numbers and weight per plant, and grain length were not significantly different between the Gu 1 × transgenic F_1_ hybrids and transgenic rice.

When compared with the Gu 1 weedy parent, shoot height, biomass, and non-shattering degree, and grain width of the Gu 1 × transgenic F_1_ hybrids were significantly higher (by 25.2%, 203.9%, 76.2%, and 3.9%, respectively). However, the percentage, number, and weight of ripened grains, and grain length were significantly lower (by 85.9%, 88.3%, 88.5%, and 5.7%, respectively), in the Gu 1 × transgenic F_1_ hybrids compared with the Gu 1 weedy parent. The number of tillers and panicles per plant, and 100-grain weight were not significantly between the Gu 1 × transgenic F_1_ hybrids and weedy rice.

### Drought tolerance of F_2_ progeny

**Figure 2:**
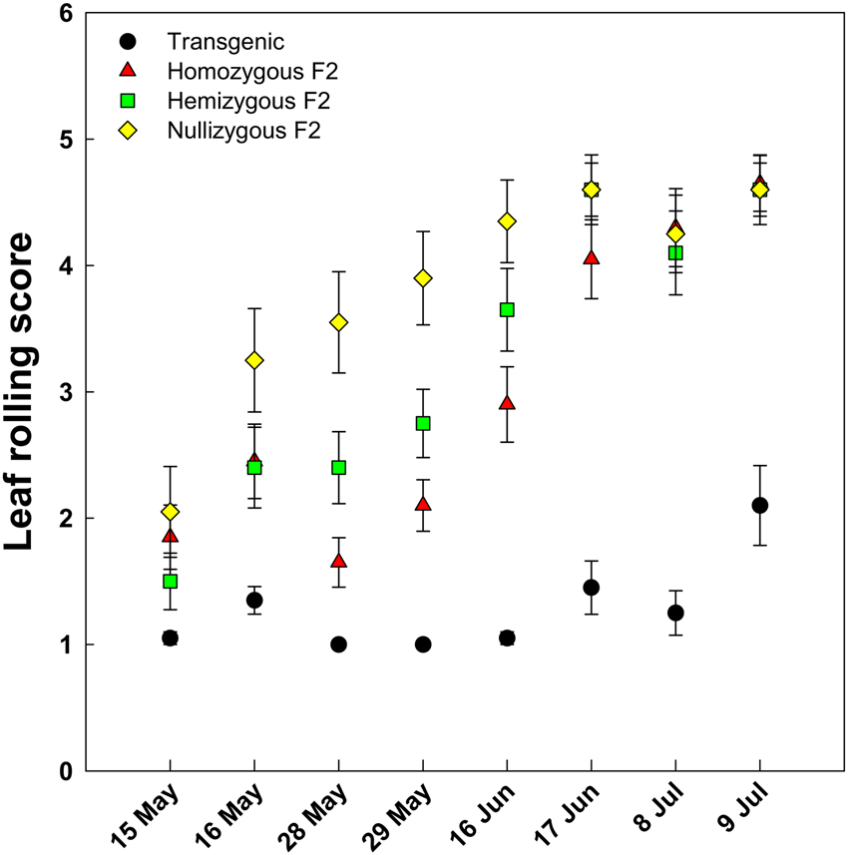
Leaf rolling score of homozygous, hemizygous, and nullizygous F_2_ progeny between *AtCYP78A7*-overexpressing transgenic rice and weedy rice (Gu 1) and their parental transgenic rice. Data are means (*n* = 20) ± standard deviations.

Compared with transgenic rice, the Gu 1 × transgenic F_2_ progeny had higher leaf-rolling scores, which indicates less tolerance to drought stress (Fig. 2). As drought stress continues, leaf rolling scores in the Gu 1 × transgenic F_2_ progeny were increased gradually, whereas those in transgenic rice remained relatively constant. During the four periods of drought stress treatment, unrolled or very slightly rolled leaves (Scores of 1 or 2) were observed in transgenic rice, whereas severe symptoms of leaf-rolling appeared in the leaves of the nullizygous F_2_ progeny. The leaves of homozygous and hemizygous F_2_ progeny had lower leaf-rolling scores compared to the nullizygous F_2_ progeny, which implies higher leaf water potential in homozygotes and hemizygotes compared with nullizygous progeny. At the final periods of drought treatment, differences in leaf-rolling score were not observed among F_2_ progeny.

### Drought-induced phenotypic changes in F_3_ progeny

**Figure 3:**
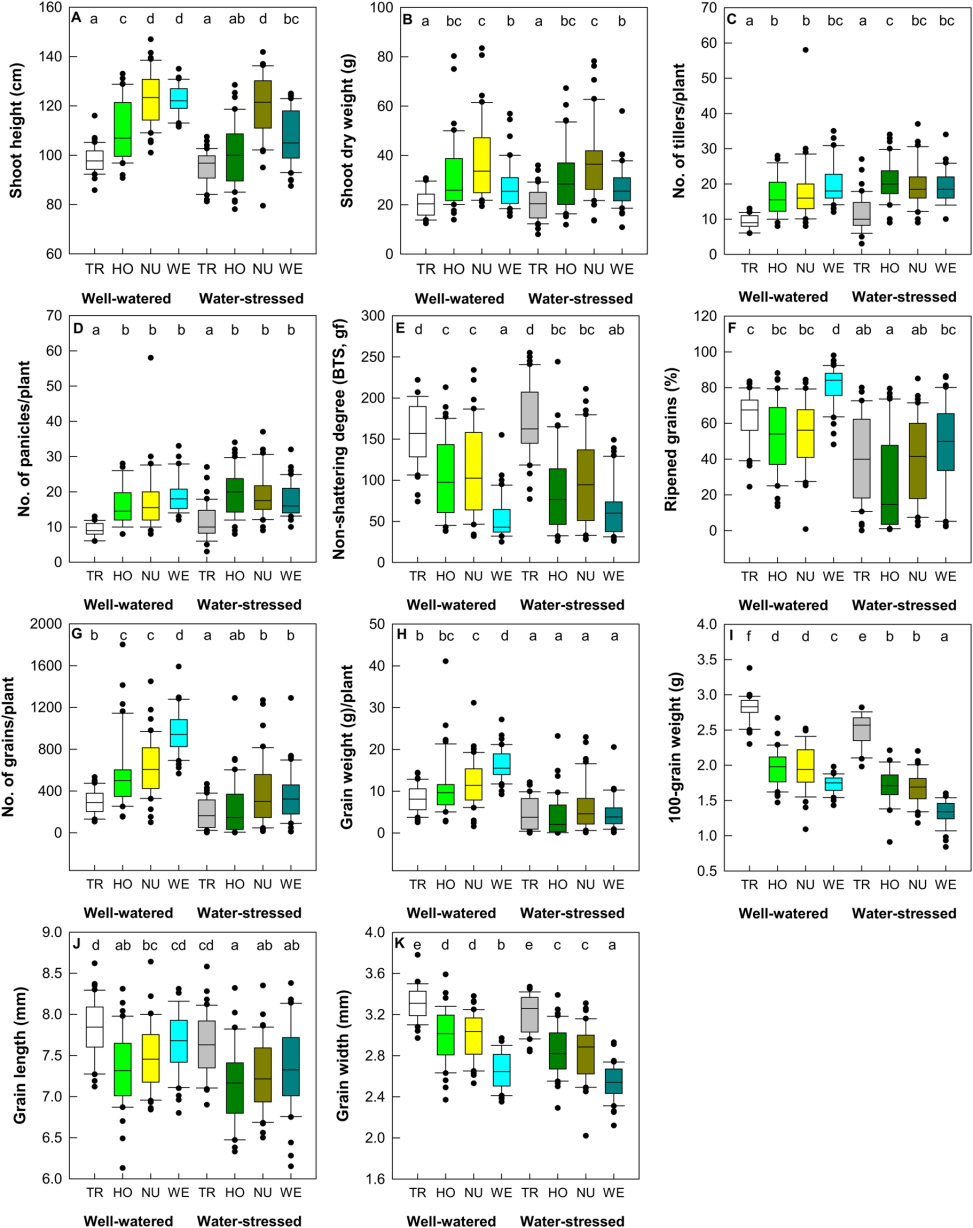
Phenotypic traits from F_3_ progeny between *AtCYP78A7*-overexpressing transgenic rice and weedy rice (Gu 1) and their parental transgenic and weedy rice under well-watered and water-stressed conditions. The boundary of the box closest to zero and farthest from zero indicates the 25th and 75th percentile, respectively, and a line within the box marks the median (*n* = 40). Whiskers above and below the box indicate the 90th and 10th percentiles, respectively and dots represent outliers. Different letters above the box indicate significant differences between means (*p* < 0.05). TR, *AtCYP78A7*-overexpressing transgenic rice; HO, homozygous F_3_ progeny between *AtCYP78A7*-overexpressing transgenic rice and weedy rice (Gu 1); NU, nullizygous F_3_ progeny between *AtCYP78A7*-overexpressing transgenic rice and weedy rice (Gu 1); WE, weedy rice (Gu 1).

Values of most phenotypic traits differ significantly according to the water condition and genotype. By comparison, the values for shoot height (7.1%) and percentage (58.8%), number (111.6%), and weight (141.1%) of ripened grains, 100-grain weight (27.2%), grain length (3.1%), and grain width (4.6%) were significantly lower for plants grown in water-deficit conditions compared with those grown in well-watered conditions; whereas those of tiller (11.1%) and panicle (10.2%) number were considerably higher in the deficit conditions (Table 2 and Fig. 3).

**Table 2:** Statistical analyses of phenotypic traits from homozygous and nullizygous F_3_ progeny following a cross of transgenic and weedy rice (Gunsanaengmi 20) and their parental transgenic and weedy rice.

Traits	Source of variation		
	Water treatment	Genotype	Water treatment × Genotype
	p-value	p-value	p-value
Shoot height (cm)^†^	<0.001	<0.001	0.010
Shoot biomass (g)^‡^	0.609	<0.001	0.995
No. of tillers/plant^†^	<0.001	<0.001	0.127
No. of panicles/plant^†^	0.004	<0.001	0.068
Ripened grains (%)^†^	<0.001	<0.001	0.041
No. of ripened grains/plant^†^	<0.001	<0.001	0.004
Weight (g) of ripened grains/plant^†^	<0.001	<0.001	0.002
100-grain weight (g)^†^	<0.001	<0.001	0.565
Non-shattering degree (BTS, gf)^†^	0.524	<0.001	0.160
Grain length (mm)^‡^	<0.001	<0.001	0.807
Grain width (mm)^†^	<0.001	<0.001	0.758

^†^ p-values are from Scheirer-Ray-Hare test.

^‡^*p*-values are from two-way ANOVA.

Under well-watered conditions, the homozygous F_3_ progeny produced 6.5% and 4.3% lower grain length compared with their parental transgenic and weedy rice, respectively (Fig. 3). The values of shoot height, number of ripened grains, 100-grain weight, non-shattering degree, and grain width in the homozygous F_3_ progeny were intermediate to those in their parental transgenic and weedy rice. Shoot biomass and number of tillers and panicles in the homozygous F_3_ progeny were similar to those in the Gu 1 weedy parent, while percentage of ripened grains and weight of ripened grains per plant in the homozygous F_3_ progeny were comparable to those in the transgenic parent. The nullizygous F_3_ progeny produced 80.9% and 37.0% higher biomass than their parental transgenic and weedy rice, respectively. The number and weight of ripened grains, 100-grain weight, non-shattering degree, and grain width in the nullizygous F_3_ progeny were in between transgenic and weedy rice. Shoot height, tiller number, panicle number, and grain length in the nullizygous F_3_ progeny were similar to those in the Gu 1 weedy parent, while percentage of ripened grains in the nullizygous F_3_ progeny were comparable to those in transgenic parent.

Under water-deficit conditions, the values of shoot height and number and weight of ripened grains in the homozygous F_3_ progeny resembled those in their parents, while the values of 100-grain weight and grain width in the homozygous F_3_ progeny exhibited mid-parent levels. Biomass, tiller number, panicle number, non-shattering degree, and grain length in the homozygous F_3_ progeny were similar to those in the Gu 1 weedy parent, while percentage of ripened grains in the homozygous F_3_ progeny were comparable to those in the transgenic parent. The nullizygous F_3_ progeny produced significantly greater shoot height (increase of 26.1% and 11.8%) and biomass (81.6% and 37.1%) than their parental transgenic and weedy rice, respectively. The percentage and number of ripened grains in the nullizygous F_3_ progeny were similar to those in their parental transgenic and weedy rice, while the 100-grain weight and grain width in the nullizygous F_3_ progeny were between the values for their parental transgenic and weedy rice. Non-shattering degree, grain length, number of tillers, panicles, and ripened grains in the nullizygous F_3_ progeny were comparable to the Gu 1 weedy parent.

A significant interaction between the watering system and genotype was detected for shoot height and percentage, number, and weight of ripened grains (Table 2). Shoot height in the transgenic parent and the nullizygous F_3_ progeny did not differ significantly between well-watered and water-deficit conditions, whereas those in the weedy parent and the homozygous F_3_ progeny were significantly decreased in response to drought stress. The percentage of ripened grains in the transgenic and weedy parents and the homozygous F_3_ progeny were clearly reduced by drought stress, while those in the nullizygous F_3_ progeny were consistent between plants grown in well-watered and water-deficit conditions.

Transgene expression was detected in homozygous F_3_ progeny by quantitative real time-PCR (Supplementary Fig. S2). The expression level of the transgene in homozygous F_3_ progeny was 67% lower than that in the transgenic parent.

## Discussion

Weedy rice has invaded and heavily infested rice fields and controlling these invaders is one of the most important issue for rice production^26^. Because it has numerous favourable ecological characteristics enabling it to outcompete cultivated rice, weedy rice may cause more serious damage to rice production in extensive regions by reducing rice yield and quality^27–29^. In particular, if weedy rice acquires a specific fitness-related trait (e.g., tolerance against biotic and abiotic stress) through gene flow derived from transgenic rice, the weedy population may increase its competitive fitness and invasiveness in an agricultural system and further induce ecological risks^2–6^. Here, we explored the transgene flow from abiotic stress-tolerant transgenic rice to their weedy relatives in a field trial and further ascertained the performance of the resultant hybrids.

The detected gene flow rate from transgenic rice to six weedy accessions varied between 0 and 0.0369%. Our results indicate that the hybridization rate between transgenic and weedy rice decreases in the weedy accession with a shorter overlap in flowering period for donor and recipient, a finding supported by previous reports^30,31^. Although the seeds of transgenic rice and weedy rice accessions were sown on different dates to synchronize flowering periods, flowering periods of every accessions were not synchronized, possibly due to their different degree of sensitivity to photoperiod^32^.

Investigating the vegetative and reproductive performance of the inter- or intra-specific hybrids is a common way of estimating fitness changes, because morphological and reproductive traits appear to be more directly correlated to the number of offspring an individual can potentially produce^33–36^. When hybridization occurs from controlled and natural crosses between transgenic glufosinate-resistant rice and red rice biotypes, F_2_ progeny exhibited a clear reduction in fitness, as measured by grain number and seed fertility compared to the parents^8^ Song *et al*.^37^ have reported that F_1_ hybrids generated between cultivated rice and its close wild relative has similar values of composite fitness as their parental species across the whole life history, implying that they could survive under natural conditions through vegetative and sexual reproduction. The F_1_ hybrids obtained from crosses between insect-resistant transgenic rice and weedy rice resulted in greater seed production and seed germination compared with their weedy parents, an observation reflecting that the expression of insect-resistance genes via transgenic introgression can improve fitness advantages in weedy populations^38^. Our recent report also suggested that the hybrids between herbicide-resistant transgenic rice and weedy rice might persist into weedy populations due to enhanced reproductive traits and herbicide resistance^6^. Here, the transgenic F_1_ hybrids overexpressing *AtCYP78A7* were significantly taller and heavier than their parental transgenic and weedy relatives. This may be explained by heterosis caused by hybridization. In contrast, the F_1_ hybrids showed a clear reduction in panicle number, grain length, and percentage, number and weight of ripened grains when compared with their weedy counterparts. Our results suggest that F_1_ hybrids could perform better than weedy rice in the field due to the increases in plant height and biomass, however, a reduction in fecundity will lead to the decrease in population size.

A heterotic effect is often observed from crossings between crops and their weedy or wild relatives, not necessarily involving transgenes^9,39^. Further, it is not yet fully understood whether the phenotypic properties of interspecific hybrids derived from gene flow from transgenic to weedy rice is an outcome of transgene movement or hybridization itself^6,40,41^. Therefore, here we compared the performance of traits in homozygous F_3_ progeny and nullizygous F_3_ progeny with those of their transgenic and weedy rice parents. Results from the present study indicate that the nullizygous F_3_ progeny produced markedly higher biomass than their transgenic and weedy rice relatives, but the height and biomass of homozygous F_3_ progeny did not increase compared to their parents under well-watered conditions. Our current findings suggest that increases in plant height and biomass of the F_1_ hybrids resulted from gene flow from *AtCYP78A7*-overexpressing transgenic to weedy rice and might be due to hybridization rather than overexpression of *AtCYP78A7*. The lower shoot height and biomass growth of homozygous F_3_ progeny compared to the nullizygous F_3_ progeny indicates the cost of continuous overexpression of transgene.

Mason *et al*.^42^ suggested that the release of transgenic insect-resistant crops creates the potential for the escape of transgenes that may provide enhanced or novel fitness-related traits via hybridization with their wild relatives. Even in the absence of exposure to glyphosate herbicide, transgenic hybrids overproducing 5-enolpyruvoylshikimate-3-phosphate synthase (*epsps*) elevated fecundity and overwintering survival/regeneration abilities than their non-transgenic controls, suggesting that the hybrids can lead to increases in the fitness of weedy populations without herbicide application^43^. The canola-weed hybrids carrying a transgene for herbivore resistance (*Bt Cry1Ac*) were larger and produced more seeds than non-transgenic plants, which were more remarkable in the presence of a biotic stressor, the diamondback moth^44^. In the present study, application of drought stress induced significant changes in the vegetative and reproductive traits of the transgenic hybrids containing *AtCYP78A7* and their parents. Particularly, the shoot height and biomass in the nullizygous F_3_ progeny greatly increased compared with their transgenic and weedy parents under water-deficit conditions. However, the performance of most traits of homozygous F_3_ progeny were similar or intermediate to those of their parents, under drought-stressed environments.

Acquisition of a certain trait in hybrids through transgene movement can lead to more favourable fitness within the weedy populations^3–6^ Chen *et al*.^29^ have expressed that the control of weedy rice may become more difficult if introgression of herbicide-resistance genes with other transgenes occurs and significantly increases the ecological fitness of weedy rice Snow *et al*.^34^ have addressed that if Bt-transgenic sunflowers are commercially released into the field, Bt transgenes will disperse from cultivated plants to natural populations of wild and weedy sunflowers and strongly reduce damage from susceptible herbivores on these plants. Here, we found that homozygous and hemizygous F_2_ progeny have higher drought tolerance, as indicated by leaf rolling index compared to the nullizygous F_2_ progeny in a pot experiment.

These evaluations were definitely required because, if commercialized, such a drought-tolerant transgenic crop would ultimately be cultivated under water-limited conditions^24^ Orians *et al*.^45^ have suggested that the performance of hybrids may be limited by low water availability, which will lower the frequency of introgression. It has also addressed that stressful agricultural environments may be more susceptible to introgression of crop alleles into wild populations^37^. Previously, we have reported that drought stress induces compositional changes in tolerant transgenic rice, which overexpress *AtCYP78A7* gene, by playing crucial roles in stress-responsive pathways (e.g., sucrose metabolism, antioxidant defenses)^24,25^. In the present study, we used transgenic rice containing a CaMV 35S promoter, which leads to the constitutive expression of transgenes. However, stress-inducible transgene expression in transgenic rice may have different outcomes compared to our study. For example Su and Wu^46^, reported that the biomass of transgenic rice containing stress-inducible promoter was significantly greater than that of transgenic rice with constitutive expression under stress environment. Therefore, the extent of stress tolerance as well as the control of transgene expression should also be considered for the study of gene flow from drought tolerant transgenic crops to weedy relatives.

In conclusion, our study demonstrates that the F_1_ hybrids of crosses between abiotic stress-tolerant transgenic rice and weedy rice had greater shoot growth but lower fecundity compared with weedy parents. Homozygous and hemizygous F_2_ progeny were more tolerant to drought than nullizygous F_2_ progeny in the pot experiment. When we compared the performance of F_3_ progeny in a rainout shelter in the field under well-watered and water-deficient conditions, we observed no increase in the fitness of homozygous F_3_ progeny. Investigations on the competitive abilities of transgenic hybrids in the mixed stands may help better elucidate the potential for hybrids to become more competitive and invade agricultural ecosystems.

## Materials and Methods

### Plant materials

Drought-tolerant transgenic japonica rice (*Oryza sativa* L.) were developed through *Agrobacterium tumefaciens*-mediated transformation^18,24^. They were derived from a rice cultivar ‘Hwayoung’ and contain *AtCYP78A7*¸ a gene that encodes a cytochrome P450 protein under the control of the cauliflower mosaic virus (CaMV) 35S promoter, *nos* terminator and hygromycin phosphotransferase (*hpt*) gene for hygromycin selection^18,24^. Seeds of a transgenic rice line (‘18A-4’) were provided by Life Sciences Research Institute, Biomedic Co. Ltd., Korea. The late Professor H.S. Suh (Yeongnam University, Korea) provided seeds of six weedy rice (*O. sativa* L., diploid) accessions, Bo 5, Ge 20, Gu 1, Hw 1, Mu 15, and Ye 3.

### Assessment of gene flow from transgenic to weedy rice

Field experiments were performed in an experimental field at the Korea Research Institute of Bioscience and Biotechnology (KRIBB), Cheongju, Korea (36°43′04″N, 127°26′07″E; elevation 37 m) as described for our previous study^6^. Based on previous field results for flowering periods of each rice line in 2011, seeds of transgenic rice and weedy accessions were sown on different dates in 2012 to synchronize the flowering periods. That is, transgenic rice was sown on 12 April; Bo 5 and Ye 3 on 2 April; Hw 1 on 4 April; Mu 15 on 8 April; Ge 20 on 15 April; Gu 1 on 14 April. Seeds were sown in a seedbed that contained commercial potting soil and grown in a greenhouse. Seven-week old seedlings were transplanted in the field with the spacing of 30 cm × 15 cm. Each row of individual weedy accessions (34 seedlings each) was positioned between two rows of transgenic rice (34 seedlings each). During the grain-filling stage, panicles of weedy accessions were bagged with nylon mesh to avoid seed loss. They were collected at 5 month after transplanting and the seeds were counted.

Resistance to hygromycin was tested to screen for hybrid seedlings between transgenic and weedy rice accessions. The seeds were surface-sterilized with a prochloraz solution for 24 h, then washed with autoclaved distilled water. They were placed in square culture dishes (size: 24.3 × 24.3 cm^2^), and 300 mL of a 0.5× MS liquid medium^47^ containing 50 mg L^−1^ hygromycin solution was added. All culture dishes were incubated in a growth chamber (25 °C, 70% relative humidity, and 16-h photoperiod) for 7 days. Seedlings with poor root and root hair growth compared with transgenic controls were considered hygromycin-sensitive. Hygromycin-resistant seedlings were transplanted into a tray filled with potting soil in a greenhouse on 30 April 2013 and then to the field on 20 May 2013. Seedlings of each rice line were transplanted in a row. The rows were 3 m long and 30 cm apart, and the distance between hills within a row was 15 cm.

**Figure 4:**
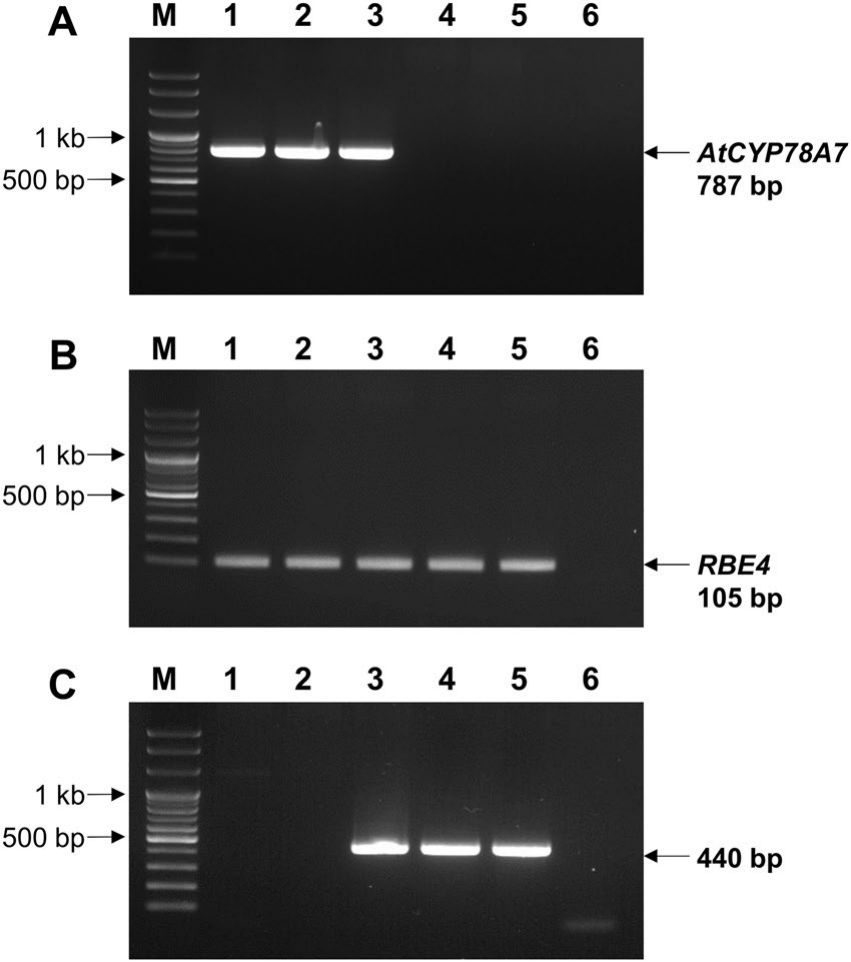
Identification of progeny following a cross between *AtCYP78A7*-overexpressing transgenic rice and weedy rice using polymerase chain reaction and gel electrophoresis. (**A**) Identification of the transgene cassette, (**B**) internal positive control, and (**C**) determination of zygosity. *M* 100 bp DNA ladder, *lane 1* transgenic rice, *lane 2* homozygous F_2_ progeny, *lane 3* hemizygous F_2_ progeny, *lane 4* nullizygous F_2_ progeny, *lane 5* weedy rice Gu 1, *lane 6* negative control (ie, no DNA).

The presence of the transgene was confirmed by PCR. Genomic DNA in 100 mg of fresh leaf tissue were extracted with a FastDNA Kit (MPBio., USA). Using a primer for the transgene cassette (AtCYP78A7-F and AtCYP78A7-R) (Table 3), we tested for the presence of the 787-bp transgene region (Fig. 4A). We also used the 105-bp RBE4 gene as an internal PCR-positive control (Fig. 4B). PCR was performed using AccuPower PCR PreMix (Bioneer, Korea) with a final volume of 20 µL that contained 1 µL of DNA template and 1 µL of each primer. The PCR amplification proceeded under the condition of an initial denaturation at 95 °C for 3 min, then 34 cycle of denaturation at 95 °C for 40 sec, annealing at 55 °C for 1 min, and extension at 72 °C for 40 sec; followed by a final extension at 72 °C for 10 min.

**Table 3:** Primer sets used to detect progeny following a cross of transgenic and weedy rice.

Primer set	Sequence (5′ to 3′)	Amplified size (bp)
**Specific to** ***AtCYP78A7*** **cassette**		
*AtCYP78A7*- F	GAT GTT TCC ACC CTA GGC TA	787
*AtCYP78A7*- R	TTT TCT TGA CGA GGG TTC TA	
**Positive control**		
*RBE4*-F	GTT TTA GTT GGG TGA AAG CGG TT	105
*RBE4*-R	CCT GTT AGT TCT TCC AAT GCC CTT A	
**Zygosity determination**		
*AtCYP78A7*- LBcfm R	AAA AGA TGA GGC CTT GAC TG	440
*AtCYP78A7*-insertion-F1	CGT GTC TGC GTC CAA GTA CA

### Zygosity determination of F_2_ progeny

Zygosity of F_2_ progeny from selfed F_1_ hybrids of transgenic and weedy rice Gu 1 was determined to compare the performance of homozygous, hemizygous and nullizygous F_2_ progeny. 100 F_2_ seeds were collected from 12 F_1_ plants (1200 seeds in total) and placed in a tray on 11 March 2014. When the seedlings reached the 3-leaf stage, their leaves were sampled. A FastDNA Kit (MPBio., USA) was used to extract genomic DNA from 100 mg of fresh tissues. Using a primer for the transgene cassette (AtCYP78A7-F and AtCYP78A7-R) (Table 3), we tested for the presence of the 787-bp transgene region in F_2_ progeny (Fig. 4A). We used the 105-bp RBE4 gene as an internal positive control (Fig. 4B). Based on samples that showed a positive band (i.e., transgene present), we determined the zygosity of the transgene. PCR was conducted with a primer for near the 5´-end (AtCYP78A7-LBcfm R) and the 3´ end of the transgene cassette (AtCYP78A7-insertion-F1). No band would be amplified for AtCYP78A7 homozygote and parental transgenic line because the extension time was too short to reveal a transgene-inserted large-sized band (ca. 5.6 kb) from 5´ to 3´. In contrast, an amplified band (440-bp between the 5´ and 3´ flanking regions) would be expected in weedy rice, F_2_ hemizygotes and nullizygotes (Fig. 4C). Therefore, both homozygous and hemizygous progenies would show positive 787-bp band but only hemizygous progenies would show 440-bp band. Both nullizygotes and weedy rice would not show 787-bp band but would show 440-bp band. PCR was performed using the method described above.

### Performance of F_1_ hybrids and F_2_ progeny

Seedlings of F_1_ hybrids that contain a transgene were marked and harvested on 21 October 2013. During the grain-filling stage, panicles from a weedy rice plant were bagged to avoid seed loss. Shoot height, shoot dry weight, number of tillers and panicles, non-shattering degree (measured as breaking tensile strength, BTS, gf), the percentage of ripened grains, number and weight of grains per plant, 100-grain weight, grain length and width of Ge 20 and Gu 1 were measured.

F_2_ progeny between transgenic rice and Gu 1, which showed the greatest rate of gene flow, were chosen to study drought tolerance. On 10 April 2014, seedlings of homozygous, hemizygous and nullizygous F_2_ progeny of transgenic and Gu 1, and transgenic rice was transplanted in Wagner pots (surface area 0.02 m^2^) filled with a mixture of commercial potting soil. A factorial experimental design with two water treatment conditions (well-watered and water-deficit) × four rice genotypes (transgenic, homozygous F_2_, hemizygous F_2_, and nullizygous F_2_) × 20 replications was utilized. The pots were randomized in 20 blocks within the greenhouse. Irrigation of one half of the pots within each block was stopped for the four periods (from 25 April to 16 May; 20 May to 29 May; 9 June to 17 June; and 1 July to 9 July, respectively) for drought treatment. A visual score of the degree of leaf rolling was recorded 8 times (15 and 16 May; 28 and 29 May; 16 and 17 June; and 7 and 8 July, respectively) using a 1 to 5 scale, where 1 = no rolling and 5 = completely rolled, according to O’Toole and Moya^48^. A lower leaf rolling score indicates higher leaf water potential of rice. Relationship between soil water contents (%) in pots and leaf rolling index of transgenic rice and hybrid progenies measured on 16 and 29 May, and 17 June is provided in Supplementary Fig. S3.

### Performance of F_3_ progeny in the rainout shelter

Performance of F_3_ progeny between transgenic rice and weedy rice Gu 1 was compared with its parental rice line in the field. Rice seeds were sown in a plastic tray containing a commercial potting soil on 8 May 2015 in a greenhouse. The seedlings were transplanted into four replicated plots in a randomized complete-block design under rainout shelters in the experimental field on 5 June 2015. A factorial experimental design of two water treatment conditions (well-watered and water-limited) and four rice genotypes (transgenic, homozygous F_3_, nullizygous F_3_, and weedy rice Gu 1). In each plot, 114 seedlings were placed on 30 cm × 15 cm spacings. Sub-surface drip irrigation with emitters of 1.49 l h^−1^ flow rate was applied to water supply system. Changes in soil moisture content (%) was monitored using EasyAG soil moisture sensors (Sentek, Australia) that were installed up to 50 cm soil depth at 30-min intervals. Water deficit stress was imposed at the beginning of the tillering stage (on 22 June 2015). For plots in the well-watered and water-deficit conditions, irrigation was automatically controlled throughout the study period to maintain soil moisture at 20% and 10%, respectively. Ten plants from each plot were harvested on 26 October 2015. Shoot height and dry weight, number of tillers and panicles, the percentage of ripened grain per panicle, the number and weight of grains, the length and width of grains, 100-grain weight and non-shattering degree were determined.

### Quantitative real time-PCR (qRT-PCR) analysis

Total RNA was isolated from rice seedlings using Biomedic RNAxzol (Biomedic Co., Ltd., Korea) according to the manufacturer’s protocol. The purified total RNA was used for the first-strand cDNA synthesis after treatment with RNase-free DNase I (Biomedic Co., Ltd., Korea). Purified total RNA (2 μg) was used for the first-strand cDNA synthesis with oligo d(T)18 and SuperiorScript II Reverse Transcriptase (Enzynomics, Korea) according to the manufacturer’s guide. Experimental samples were evaluated in duplicate and qRT-PCR reactions for each were run in triplicate. PCR was conducted using a LightCycler 480 II Real Time PCR Instrument (Roche Diagnostics GmbH) in a total volume of 20 μL containing 4 μL of cDNA (<100 ng), 0.4 μL each of forward and reverse primers (10 pmol μL^−1^), and 10 μL of TOPreal qPCR 2× PreMIX (Enzynomics, Korea). The conditions for PCR amplification were as follows: 5 min for initial denaturation at 95 °C, and 55 cycles of 15 sec at 95 °C, 20 sec at 60 °C and 20 sec at 72 °C. The comparative C_T_ method, also referred to as the 2^-ΔΔCT^, was used to analyze the relative gene expression of target gene^49^. Target gene expression was normalized with *OsACT1*, a rice housekeeping gene. A pair of primers; 78A7RT-F (5′- GGTACGACGGTTCGAGTGGGGTCAGGA-3′) and 78A7RT-R (5′-GTTGTCGAGAGGTATGAATTGCAGA-3′), were designed for the expression analysis of the target gene, *AtCYP78A7*. OsACT1F (5′-ATGTTCCCTGGCATTGCTGA-3′) and OsACT1R (5′-CGGCGATAACAGCTCCTCTT-3′) were designed for the expression analysis of rice housekeeping gene, *OsACT1*.

### Data analyses

Data were analyzed using Excel with the Real Statistics Using Excel add-in (Release 6.2.2)^50^. Data were tested for normality and homoscedasticity using the Shapiro-Wilk and Levene’s tests, respectively. Non-normally distributed data were log- or square root-transformed to fit normal distributions.

The overall effect of rice genotype on performance of F_1_ hybrids was evaluated by one-way analysis of variance (ANOVA). Statistically significant differences between means were identified by Tukey’s honestly significant difference (HSD) test (*P* < 0.05). When the data did not meet the assumption for ANOVA despite the data transformation, the effect of rice genotype was analyzed with Kruskal-Wallis test and statistically significant differences between means were identified by Dunn’s test.

The overall effect of rice genotype and watering condition on shoot biomass and grain length of F_3_ progeny were evaluated by two-way ANOVA and statistically significant differences between means were identified by Tukey’s honestly significant difference (HSD) test (*P* < 0.05). The effect of rice genoypte and watering condition on the other traits of F_3_ progeny were evaluated by Scheirer-Ray-Hare Test and statistically significant differences between means were identified by multiple Mann-Whitney tests with Bonferroni correction.

## Supplementary information

Supplementary information.

## Data Availability

All relevant data are available within the manuscript.
