# Supplementary material for: Performance of hybrids between abiotic stress-tolerant transgenic rice and its weedy relatives under water-stressed conditions
Source: Sci Rep. 2020 Jun 9;10:9319. doi: 10.1038/s41598-020-66206-3 (PMC7283212; doi:10.1038/s41598-020-66206-3)
Supplement: Supplementary file 1 — Supplementary information. [file 41598_2020_66206_MOESM1_ESM.docx]

**Supplementary material**

**Performance of hybrids between abiotic stress-tolerant transgenic rice and its weedy relatives under water-stressed conditions**

Kyong-Hee Nam^1^, Do Young Kim^2^, Ye Seul Moon^2^, In Soon Pack^2^, Soon-Chun Jeong^2^, Ho Bang Kim^3^, Chang-Gi Kim^2 *^

^1^*LMO research team, National Institute of Ecology, Seocheon 33657, Republic of Korea*

^2^Bio-Evaluation Center, Korea Research Institute of Bioscience & Biotechnology, Cheongju 28116, Republic of Korea

^3^Life Sciences Research Institute, Biomedic Co., Ltd., Bucheon 14548, Republic of Korea


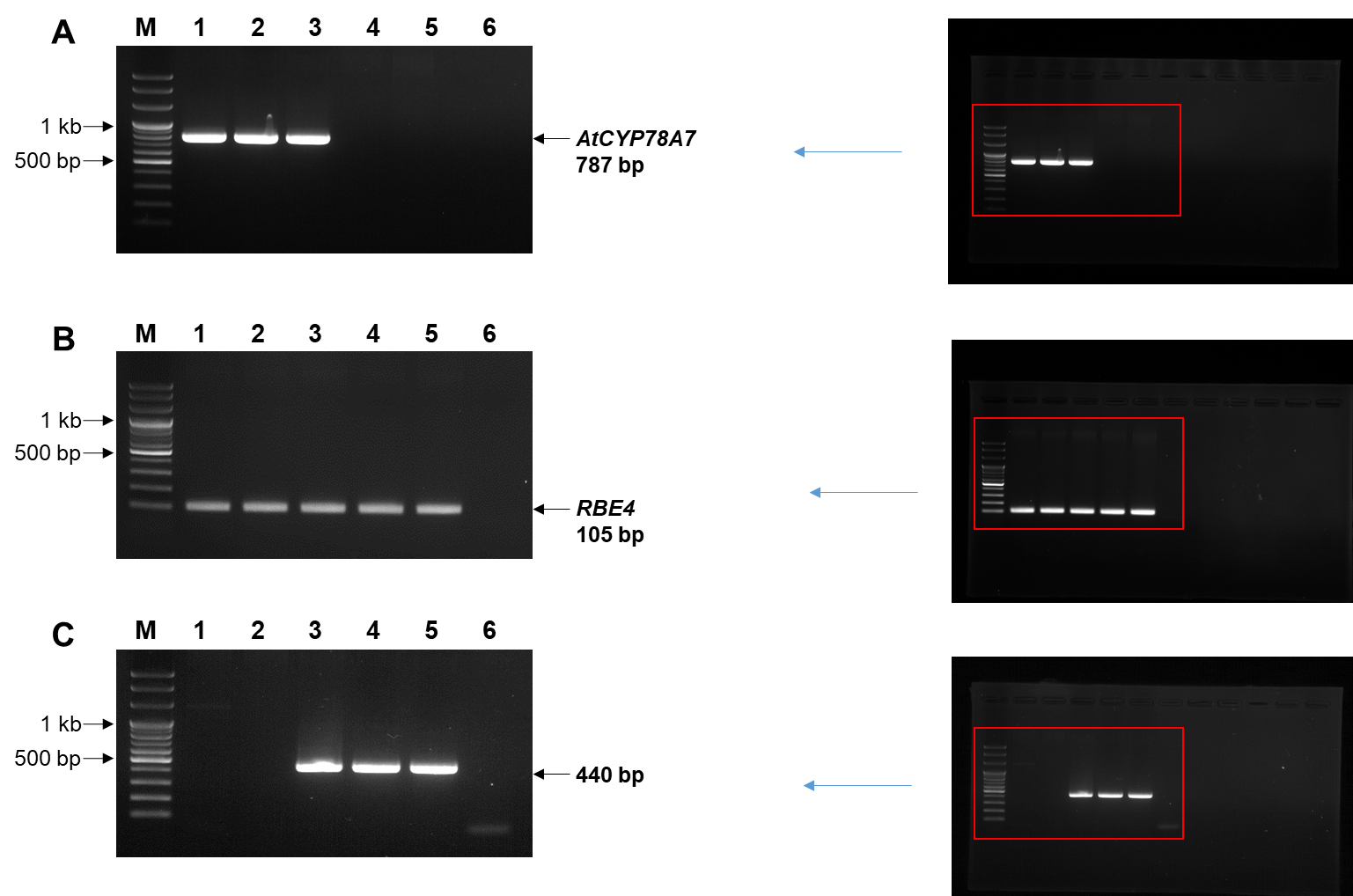


**Raw data**

**Fig S1. Full-length gels used to generate Fig. 1.**

**
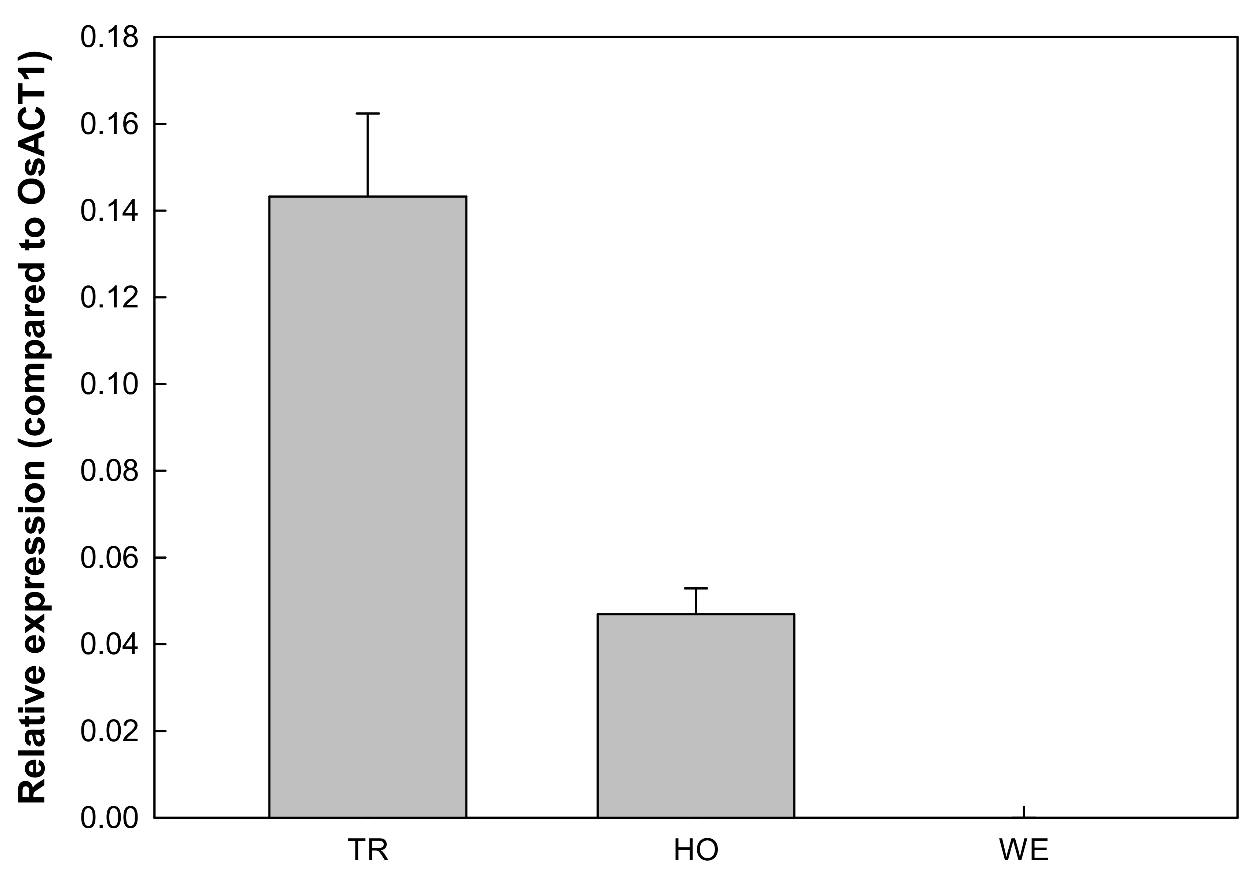
**

**Fig. S2. Relative expression of *AtCYP78A7* gene in *AtCYP78A7*-overexpressing transgenic rice (TR), weedy rice (WE, Gu 1), and homozygous F_3_ progeny (HO) between transgenic rice and weedy rice. Expression of transgene was normalized with rice *OsACT1* gene. Data are means ± standard errors.**

**Fig. S3. Relationship between soil water contents (%) in pots and leaf rolling index of transgenic rice and hybrid progenies measured on 16 and 29 May, and 17 June.**
